# Whole-genome sequencing and genomic analysis of four *Akkermansia* strains newly isolated from human feces

**DOI:** 10.3389/fmicb.2024.1500886

**Published:** 2024-12-16

**Authors:** Wenjing Lu, Biqing Zha, Jie Lyu, Chenxi LingHu, Jing Chen, Sisi Deng, Xiangling Zhang, Liang Li, Guoqing Wang

**Affiliations:** ^1^West China School of Public Health and West China Fourth Hospital, Sichuan University, Chengdu, China; ^2^Jiujiang Center for Disease Control and Prevention, Jiujiang, China; ^3^Microbiome Research and Application Center, BYHEALTH Institute of Nutrition and Health, Guangzhou, China; ^4^Chengdu East New District Public Health Center, Chengdu, China; ^5^Hunan Provincial Center for Disease Control and Prevention, Changsha, China

**Keywords:** *Akkermansia*, whole-genome sequencing, genomic analysis, genomic diversity, probiotics

## Abstract

**Background:**

Numerous studies have demonstrated that *Akkermansia* is closely associated with human health. These bacteria colonize the mucus layer of the gastrointestinal tract and utilize mucin as their sole source of carbon and nitrogen. *Akkermansia* spp. exhibit potential as probiotics under specific conditions. However, the gene accumulation curve derived from pan-genome analysis suggests that the genome of *Akkermansia* strains remains open. Consequently, current genome mining efforts are insufficient to fully capture the intraspecific and interspecific characteristics of *Akkermansia*, necessitating continuous exploration of the genomic and phenotypic diversity of new isolates.

**Methods:**

Based on this finding, we sequenced, assembled, and functionally annotated the whole genomes of four new human isolates from our laboratory: AKK-HX001, AKK-HX002, AKK-HX003, and AKK-HX004.

**Results:**

Phylogenetic analysis revealed that all four isolates belonged to the AmII phylogroup, whereas the type strain DSM 22959 is classified within the AmI phylogroup. Moreover, 2,184 shared homologous genes were identified among the four isolates. Functional annotation using the COG, KEGG, and CAZy databases indicated that the functional genes of the four isolates were primarily associated with metabolism. Two antibiotic resistance genes were identified in AKK-HX001 and AKK-HX002, while three resistance genes were detected in AKK-HX003 and AKK-HX004. Additionally, each of the four isolates possessed two virulence genes and three pathogenicity genes, none of which were associated with pathogenicity. The prediction of mobile genetic elements indicated unequal distributions of GIs among the isolates, and a complete CRISPR system was identified in all isolates except AKK-HX003. Two annotated regions of secondary metabolite biosynthesis genes, both belonging to Terpene, were detected using the antiSMASH online tool.

**Conclusion:**

These findings indicate that the four *Akkermansia* isolates, which belong to a phylogroup distinct from the model strain DSM 22959, exhibit lower genetic risk and may serve as potential probiotic resources for future research.

## 1 Introduction

The intestinal tract possesses the richest microbiota in the body: the intestinal microbiota, which is closely related to the development of human diseases (Hou et al., 2022; Ruigrok et al., 2023). Rinninella et al. (2019) concluded that the composition of the gut microbiota is unique to each individual and varies at all stages of life. With the rise of large-scale sequencing technologies, the composition and function of the gut microbiota are gradually being decoded, and more and more links between diseases and the gut microbiota are being characterized. Hence, it is possible to design personalized drugs for specific diseases that are targeted to an individual’s specific gut microbiota (Singh and Natraj, 2021). A subset of microorganisms derived from human commensals have potential to be the next-generation probiotics (NGPs), which attract increasing attention. With the deepening of multi-faceted research, it has been found that the adaptability of these strains to the intestinal environment allows them to produce bioactive compounds and have a positive impact on gut health, immune function, and metabolism, which may not be possible with traditional probiotics (Lalowski and Zielińska, 2024). Their ability to target specific health problems may include the production of bioactive compounds, the modulation of immune responses, and competitive interactions with pathogens, all of which may give NGPs an advantage in targeted therapies for chronic diseases (Abouelela and Helmy, 2024; Lalowski and Zielińska, 2024).

*Akkermansia muciniphila* (*A. muciniphila*), a representative of the *Verrucomicrobia* phylum, was first isolated from human feces in 2004 by Derrien et al. (2004), is a focal point of such studies due to its ability to colonize the intestinal mucus layer and influence host physiology (Derrien et al., 2004; Derrien et al., 2017). Numerous studies have linked *A. muciniphila* to beneficial health outcomes, including its role in reducing metabolic disorders, regulating the immune response, and maintaining gut barrier integrity (Everard et al., 2013; Depommier et al., 2019). For example, its abundance correlates positively with lower obesity risk, reduced inflammation in inflammatory bowel disease (Pittayanon et al., 2020), and improved glucose metabolism in diabetic patients (McMurdie et al., 2022). Additionally, novel associations with familial Mediterranean fever and tumor modulation have been reported (Pepoyan, 2024; Pepoyan et al., 2024; Zhao et al., 2023). However, recent studies present a contrasting perspective. Research by Gleeson et al. (2024) and Pereira et al. (2024) suggests that *A. muciniphila* may exacerbate immune nephropathy and induce inflammation in the absence of dietary fiber. These findings emphasize the importance of context and strain specificity in evaluating its probiotic potential. Such conflicting results underscore the urgent need for strain-level functional studies to clarify safety profiles and therapeutic applications.

The ability to isolate and characterize *Akkermansia* strains has significantly expanded in recent years (Becken et al., 2021; Geerlings et al., 2021). Improved culturing methods have revealed substantial inter- and intra-species genomic variability, which influences host adaptation and functional traits (Guo et al., 2017; Kirmiz et al., 2020; Yang et al., 2020; Becken et al., 2021). Pan-genome analyses indicate that *Akkermansia* has an open genome structure (Guo et al., 2017), with new genetic features discovered in every additional strain isolated. This highlights the need to continue isolating strains and analyzing their phenotypic diversity to fully understand their roles in the microbiota. Developing an *Akkermansia* gene library could aid in identifying functional traits, enabling targeted applications in precision medicine. Furthermore, strain-specific adaptations to hosts and geographic regions suggest the potential for designing probiotics tailored to specific populations.

In this study, four *Akkermansia* isolates from the intestinal tract of Chinese infants and young children, previously isolated in the laboratory, were subjected to whole genome sequencing to analyze and characterize their potential functional features, safety, and horizontal gene transfer ability at the gene level. This study aims to provide a theoretical basis for subsequent research on the functional and beneficial effects of *Akkermansia*, contribute new insights into the regional and population diversity of *Akkermansia* strains, and use genomic data to guide the selection of practical strains.

## 2 Materials and methods

### 2.1 Microorganisms and culture conditions

*Akkermansia muciniphila* (*A. muciniphila*, DSM22959) was generously provided by Prof. Liu Li, Nanjing Agricultural University. The 4 *Akkermansia* strains were isolated from the feces of infants provided by Sichuan Provincial Maternity and Child Health Care Hospital using a conventional isolation process. Briefly, the fecal glycerol tube was thawed in a 37°C water bath, diluted tenfold with saline, and cultured under anaerobic conditions for 5 days at 37°C in mucin liquid medium supplemented with vancomycin (6 μg/mL) and kanamycin (12 μg/mL) for bacterial enrichment. During the growth process, 100 μL of a uniformly turbid bacterial solution was taken from the anaerobic tube, diluted tenfold to 10^−5^, and 100 μL of the diluted solution was spread onto BHI agar plates containing vancomycin and kanamycin for isolation and culture. The plates were incubated anaerobically at 37°C for 1–2 weeks, with PCR detection conducted in parallel. Single colonies suspected of containing the target bacteria were isolated and purified through plate streaking and BHI liquid medium enrichment. The DNA of the isolates was then extracted, and 16S rRNA sequencing was performed after PCR amplification using universal primers 1492R and 27F. The sequencing results were uploaded to GenBank for comparison, confirming that the 16S rRNA sequences of the 4 isolates showed 100% similarity to those of *Akkermansia*, verifying their classification within the genus. The strains were designated as AKK-HX001, AKK-HX002, AKK-HX003, and AKK-HX004. The bacterial solutions were preserved at −80°C with the addition of 30% glycerol in a 1:1 ratio.

The preserved strains were recovered by streaking on BHI agar plates (1 week), activated, and multiplied in BHI liquid medium (48 h), with all processes carried out anaerobically at 37°C.

### 2.2 Extraction of DNA, library construction, and whole-genome sequencing

Bacterial cells were harvested by centrifugation of the logarithmic growth phase of the bacterial solution at 12,000 r/min for 5 min. DNA extraction was performed using the TIANamp Bacteria DNA Kit, according to the manufacturer’s instructions. DNA quantity and purity were measured by the NanoDrop 2000 (Thermo Fisher Scientific).

Library preparation was performed using the NEBNext^®^Ultra^™^ II DNA Library Prep Kit for Illumina (NEB, United States, Catalog #: E7370L), with 0.2 μg of total DNA per sample. Genomic DNA samples were fragmented by sonication, and 350 bp fragments were selected for end-polishing, A-tailing, and ligation to full-length adapters, followed by PCR amplification. Agencourt AMPure XP (Beverly, United States) purified the PCR products. Sequencing was performed on the Illumina Miseq PE300 (Novogene, China, Peking) platform after the evaluation of library construction.

### 2.3 Genome assembly and prediction

Quality control and filtering of whole genome sequencing results were performed using Fastp (Chen et al., 2018). *De novo* assembly was performed using SPAdes (Prjibelski et al., 2020), followed by genome annotation using Prokka (Grant et al., 2023), both of which are available sources. Predictions and mapped genomic circular maps were created using the CGView online database (Stothard et al., 2019). Homology analysis was performed using OrthoFinder (Emms and Kelly, 2019) to obtain the gene family classification of the 4 *Akkermansia* isolates and their respective numbers.

### 2.4 Phylogenomic analysis and comparison of average nucleotide identity

Fifteen human *Akkermansia* isolates of known phylogroups as well as the model strain DSM22959 were selected and their genome sequences were downloaded from NCBI. The 16S rRNA sequences were extracted from the 20 whole-genome sequences using Barrnap, imported into MEGA11 (Tamura et al., 2021) for comparison and clipping, and selected the “Maximum Likelihood Tree” approach, with Bootstrap set to 1,000 for phylogenetic tree construction. Phylogenomic analysis based on single-copy core genes was performed using GToTree (Lee, 2019), and the evolutionary tree was landscaped using the ITOL online website (Letunic and Bork, 2024). FastANI (Jain et al., 2018) was used to calculate the average nucleotide identity (ANI) between the 16 sequences and the 4 isolate genomes at two intervals.

### 2.5 Functional annotation

In order to recognize the function of the predicted genes, the web annotation tool eggNOG-mapper (Cantalapiedra et al., 2021) was used to blast with the Clusters of Orthologous Groups of proteins (COG) database. In parallel, an analysis was conducted using KofamKOALA (Aramaki et al., 2020) to compare the function and composition of the 4 isolates’ biological systems by Kyoto Encyclopedia of Genes and Genomes (KEGG) database annotation. Carbohydrate-Active enZYmes Database (CAZy) database (Drula et al., 2022) annotation was performed by importing the amino acid sequences of predicted genes into the DbCAN3 website (Zheng et al., 2023). Secondary metabolite gene clusters were identified by antiSMASH (Blin et al., 2023).

### 2.6 Genomic safety assessment based on CARD, VFDB, and PHI databases

To assess the safety of the 4 *Akkermansia* isolates at the genetic level, their antibiotic resistance, virulence factors, and pathogenicity genes were analyzed separately by blasting against The Comprehensive Antibiotic Resistance Database (CARD) (Alcock et al., 2023), Virulence Factor Database (VFDB) (Liu et al., 2022), and Pathogen-Host Interactions database (PHI) (Urban et al., 2022), where CARD is based on “the perfect and strict hits” select criteria, VFDB and PHI employed the results with the cut-off value at >70% identity and optimal comparison results for further analysis.

### 2.7 Analysis of mobile genetic elements

The 4 isolates were predicted to mobile genetic elements (MGEs) after genome rearrangement using MAVUE software (Darling et al., 2004). In this study, the MGEs we analyzed included genomic islands (GI) and the Clustered Regularly Interspaced Short Palindromic Repeats-Cas (CRISPR-Cas) system, which were predicted by IslandView 4 (Bertelli et al., 2017) and CRISPRCas Finder (Couvin et al., 2018), respectively.

### 2.8 Statistical analysis

If not specifically indicated, the visualization of the data was achieved through Origin (Version 2021, OriginLab Corporation, Northampton, MA, United States).

## 3 Results

### 3.1 General genomic characteristics of the isolates

**Figure 1:**
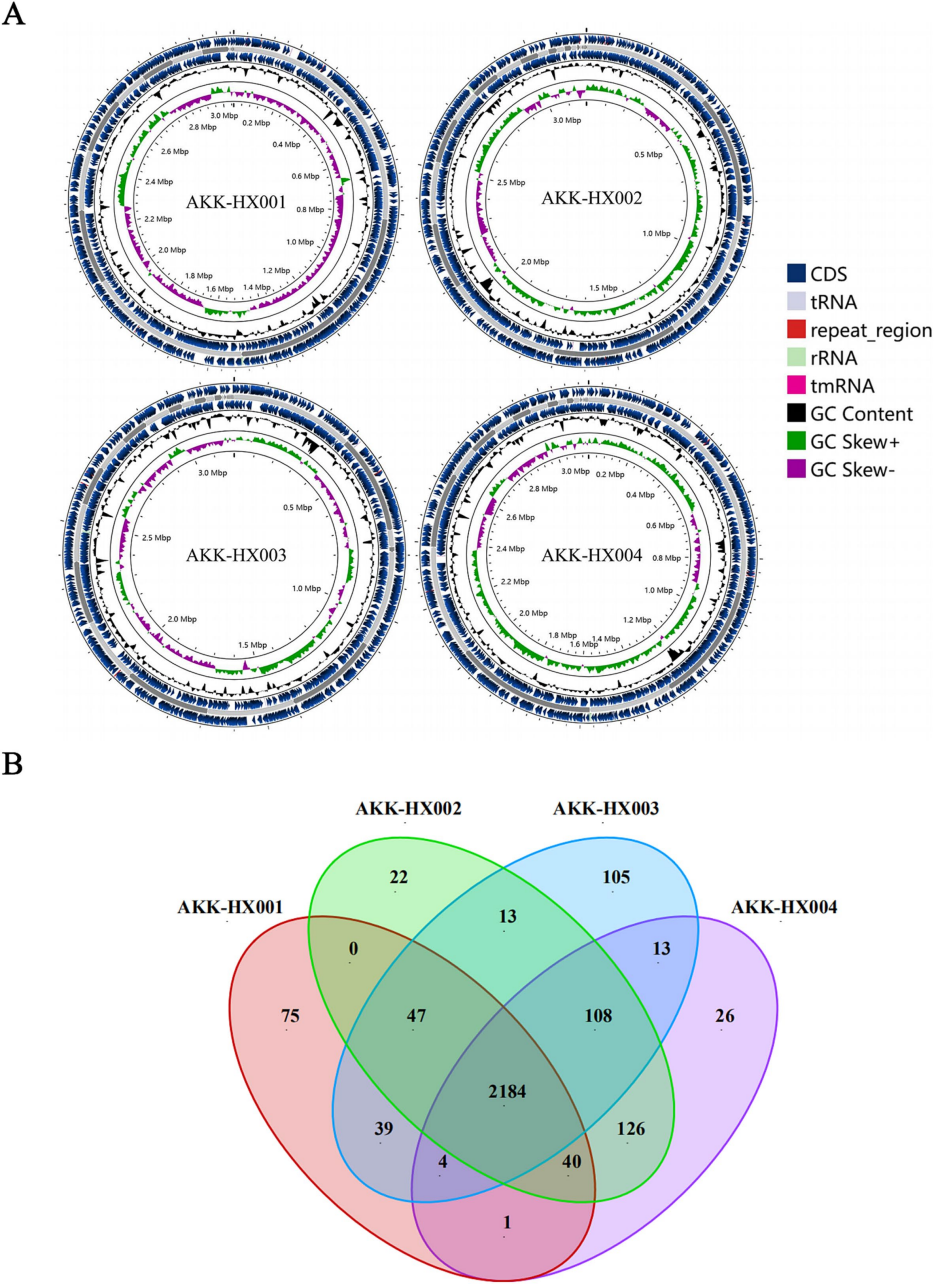
Circular maps of AKK-HX001, AKK-HX002, AKK-HX003, AKK-HX004. From the inside out, the order is GC Skews (GC Skew+: green, GC Skew−: purple), GC Content (black), CDS (blue), tRNA, rRNA, repeat_region, and tmRNA **(A)**. The Venn diagram was based on the homologous genes and unique genes of the 4 *Akkermansia* isolates (**B**).

The genome size, GC content, protein-coding sequences (CDS), and repeat_regions of the 4 *Akkermansia* isolates generally exceeded those of the model strain, and rRNA counts were lower than the type strain. In contrast, the amounts of tRNA were 52, 54, 51, and 53, respectively, which were close to the DSM22959 (54), and all of them encompassed 1 tmRNA, distinctively. The details were displayed in Table 1 below and the genome circular diagram was shown in Figure 1A. Homology analysis revealed 2,184 homologous genes in the genomes of the 4 *Akkermansia* isolates, with 75, 22, 105, and 26 unique genes, respectively (Figure 1B). COG functional annotation of genes specific to the 4 isolates revealed that only 10.53% (24/228) of the genes were assigned to the corresponding functional categories and were mainly related to information storage and processing (8/24), with the majority of the genes encoding hypothetical proteins for which the specific functions they exercise are not known (11/24).

**Table 1:** General characterization of the genomes of 4 *Akkermansia* isolates.

Class definition	AKK-HX001	AKK-HX002	AKK-HX003	AKK-HX004	DSM22959
Genome size (Mb)	3.21	3.28	3.28	3.51	2.67
GC percent (%)	58.18	57.82	57.78	57.72	55.80
CDS	2,474	2,653	2,658	2,587	2,184
Number of repeat_region	4	5	4	4	1
Number of rRNA	3	3	3	3	9
Number of tRNA	52	54	51	53	54
Number of tmRNA	1	1	1	1	1

### 3.2 Phylogenetic analysis

#### 3.2.1 16S rRNA phylogenetic tree analysis and phylogenomic analysis

**Figure 2:**
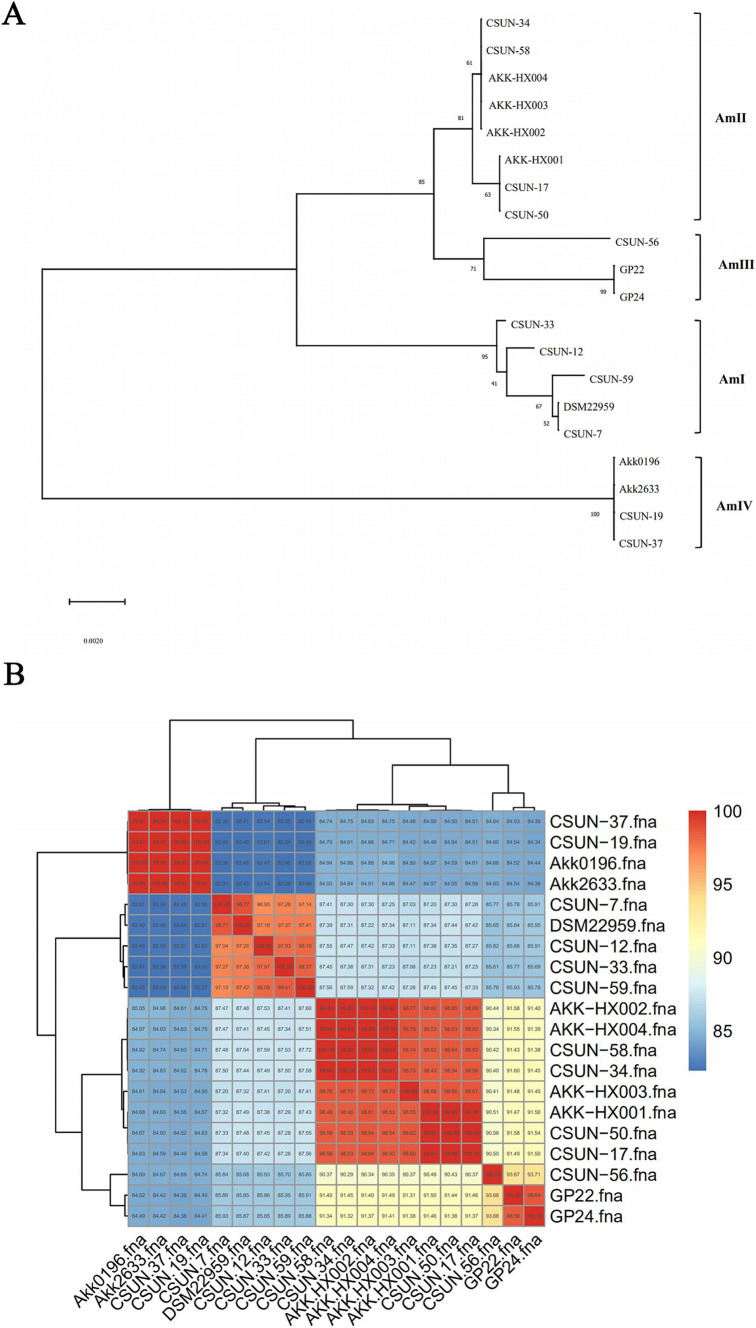
Phylogenetic tree based on 16S rRNA. The value of each branch node in the phylogenetic tree represents the percentage of bootstrap values from 1,000 repetitions **(A)**. The heatmap was based on the ANI values. The values shown on the heatmap indicate the similarity between each of the two given genomes **(B)**.

Previously, Kirmiz et al. (2020) classified the genus *A. muciniphila* into four phylogenetic groups based on the research of Guo et al. (2017). To clarify the phylogroup classification of the 4 *Akkermansia* isolates from our laboratory, 15 human *Akkermansia* isolates with known phylogroup classifications and DSM22959 were selected. Their whole-genome sequences were downloaded from the NCBI, and their 16S rRNAs were extracted to construct the phylogenetic tree (Figure 2A). The 4 *Akkermansia* isolates were subsumed into the AmII phylogroup, while DSM22959 belonged to the AmI phylogroup by contrast. The results showed that AKK-HX001 belonged to a different evolutionary branch from AKK-HX002, AKK-HX003, and AKK-HX004, suggesting that AKK-HX001 may be more distantly related to the other three strains. Phylogenomic analysis based on single-copy core genes also yielded consistent results (Supplementary Figure S1). This was confirmed by a phylogenetic tree built based on ANI (Figure 2B). The ANI values between the 4 isolates and DSM22959 were 87.49, 87.48, 87.32, and 87.41%, respectively, while the ANI values among the 4 isolates were as high as 98.53–99.93%.

### 3.3 Genome functional classification

#### 3.3.1 COG annotation

**Figure 3:**
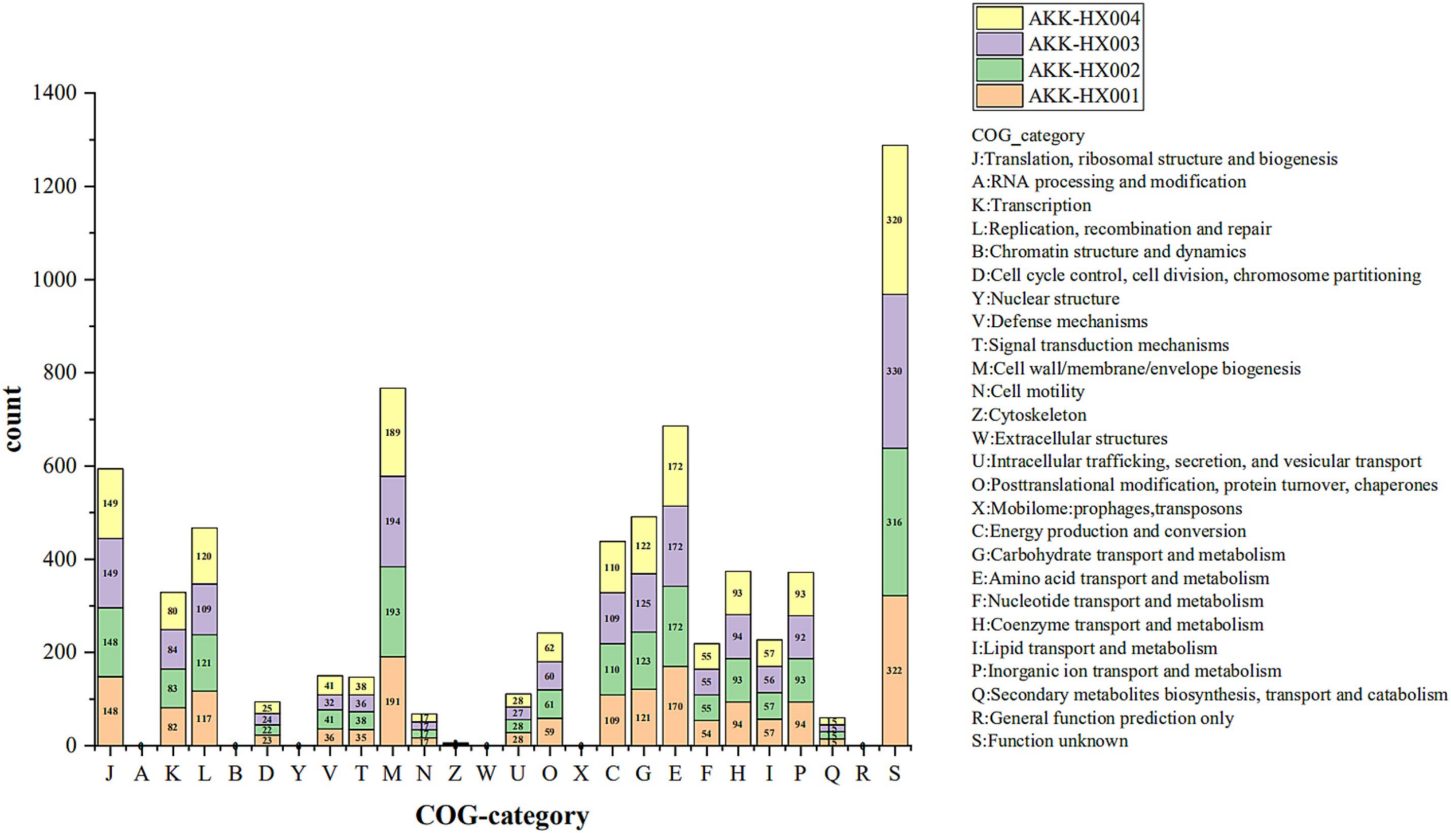
Stacked histogram of COG functional classification of 4 *Akkermansia* isolates. COG functions were categorized into 4 main groups: Information Storage and Processing: J-B, Cellular Processes and Signaling: D-X, Metabolism: C-Q, Poorly Characterized: R, S.

COG includes 4 major classifications, information storage and processing, cellular processes and signaling, metabolism, and category unknown, which are also subdivided into 26 functional categories. Four *Akkermansia* isolates were predicted to have 1,772, 1,788, 1,782, and 1,788 functional protein genes annotated into 20 functional categories by COG analysis, respectively. Among them, the higher number of functional genes was category M (Cell wall/membrane/envelope biogenesis), category E (Amino acid transport and metabolism), category J (Translation, ribosomal structure and biogenesis), category G (Carbohydrate transport and metabolism), category L (Replication, recombination and repair), and category C (Energy production and conversion) (Figure 3). Notably, AKK-HX001 lacked the category Z (Cytoskeleton) functional gene, while the other three strains annotated two genes of category Z.

#### 3.3.2 KEGG annotation

**Figure 4:**
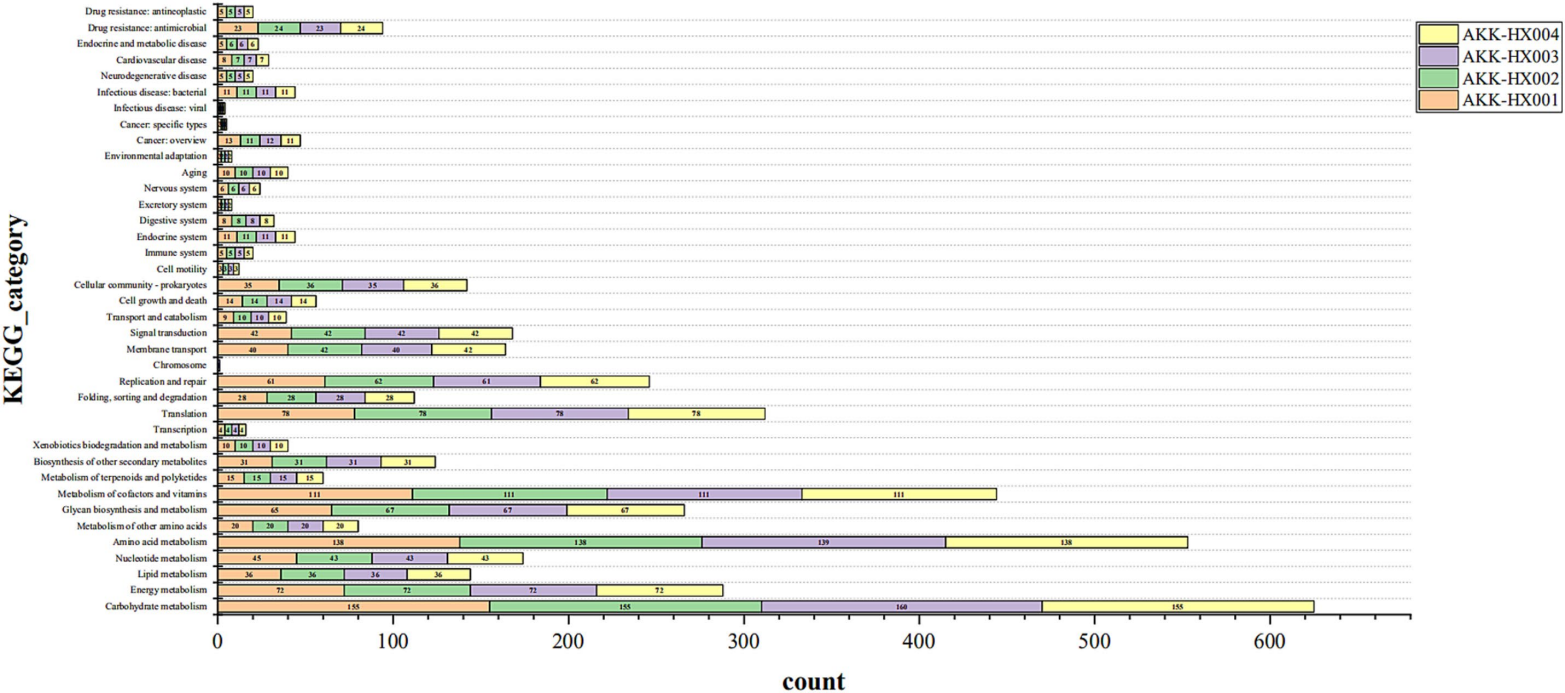
The stacked bar of KEGG functional classification of 4 *Akkermansia* isolates. Metabolism: Carbohydrate metabolism, Energy metabolism, Lipid metabolism, Nucleotide metabolism, Amino acid metabolism, Metabolism of other amino acids, Glycan biosynthesis and metabolism, Metabolism of cofactors and vitamins, Metabolism of terpenoids and polyketides, Biosynthesis of other secondary metabolites, Xenobiotics biodegradation and metabolism. Genetic Information Processing: Transcription, Translation, Folding, Sorting and degradation, Replication and repair, Chromosome. Environmental Information Processing: Membrane transport, Signal transduction. Cellular Processes: Transport and catabolism, Cell growth and death, Cellular community – Prokaryotes, Cell motility. Organismal Systems: Immune system, Endocrine system, Digestive system, Excretory system, Nervous system, Aging, Environmental adaptation. Human Diseases: Cancer: Overview, Cancer: Specific types, Infectious disease: Viral, Infectious disease: Bacterial, Neurodegenerative disease, Cardiovascular disease, Endocrine and metabolic disease, Drug resistance: Antimicrobial, Drug resistance: Antineoplastic.

KEGG pathway annotation of the predicted protein-coding genes of the 4 *Akkermansia* isolates resulted in 1,129, 1,132, 1,135, and 1,132 genes being included in the annotation of 203, 204, 204, and 204 metabolic pathways, respectively. These genes belonged to six major categories in the KEGG pathway first-level classification: metabolism, genetic information processing, environmental information processing, cellular processes, organismal systems, and human diseases, and were mainly annotated to the first two major categories. Each of the 37, 37, 38, and 37 metabolic pathways was annotated in the second-level classification (Figure 4). AKK-HX003 had more genes annotated to “metabolism,” especially “carbohydrate metabolism,” which suggested that AKK-HX003 may have a stronger ability to utilize and degrade carbohydrates. AKK-HX003 had one gene annotated to the Chromosome, while the other three strains did not have. The coding genes that were annotated to metabolism were the most numerous and were mainly related to carbohydrate metabolism, amino acid metabolism, metabolism of cofactors and vitamins. Next up was genetic information processing, and the coding genes annotated to translation, replication and repair processes were mainly involved. In terms of environmental information processing, the coding genes were mostly related to membrane transport and signal transduction. As for human diseases, the coding genes were significantly associated with antibiotic resistance.

#### 3.3.3 CAZy annotation

**Figure 5:**
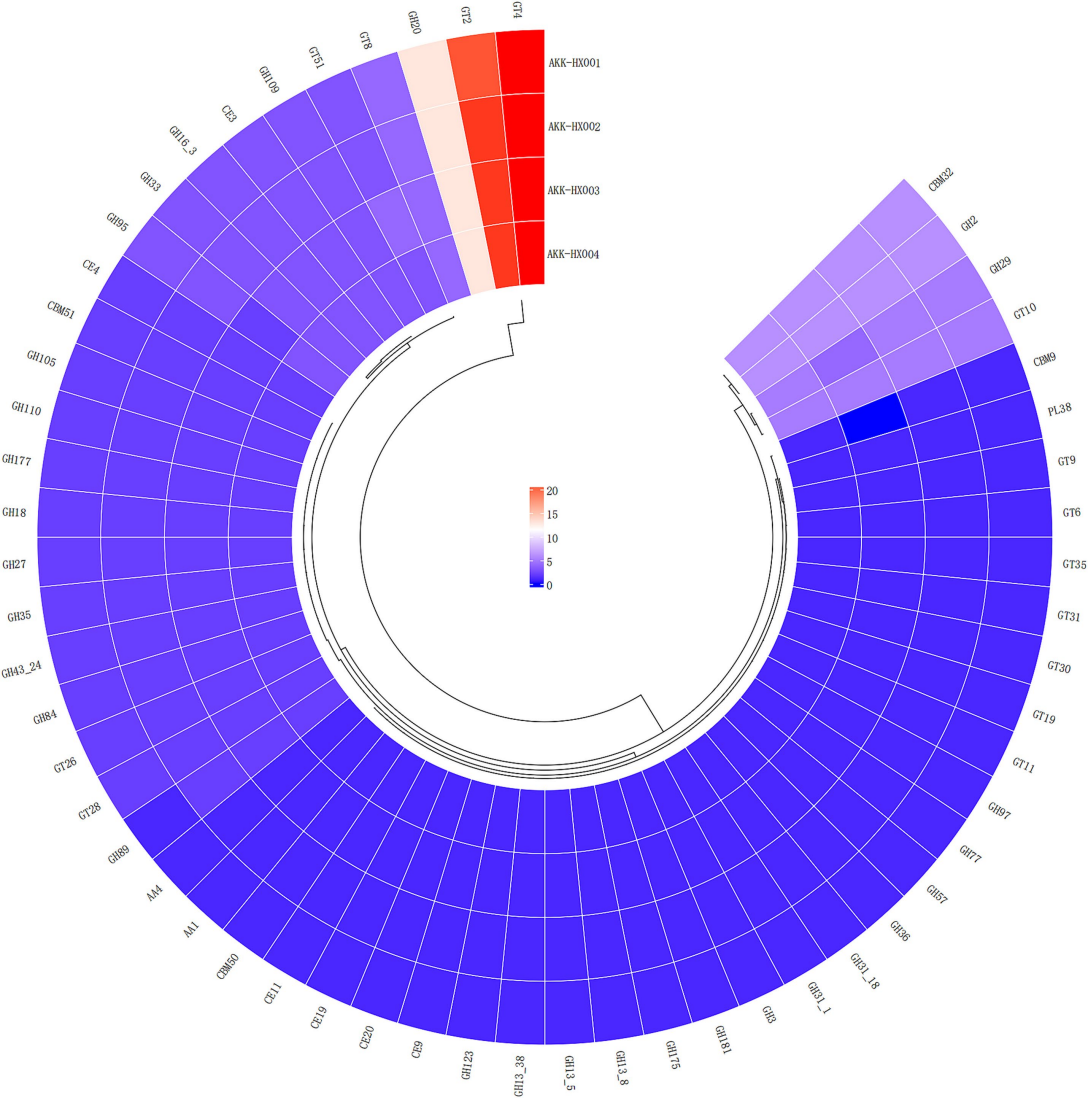
Cyclic thermogram of CAZy annotations of the 4 *Akkermansia* isolates.

The 4 *Akkermansia* isolates, with 146, 149, 147, and 149 coding genes, were annotated to six carbohydrate-active enzyme families, respectively. These included 2 AAs (Auxiliary Activities), 4 CBMs (Carbohydrate-Binding Modules) (AKK-HX003 contained only three and lacked CBM9), which has only been found in xylanases with the ability to bind cellulose so far (Selvaraj et al., 2010), 6 CEs (Carbohydrate Esterases), 29 GHs (Glycoside Hydrolases), 14 GTs (GlycosylTransferases), and 1 PLs (Polysaccharide Lyases) (Figure 5). The largest number of genes was assigned to the GHs and GTs families, and the results of the annotations for the AAs and PLs were consistent, as cataloged in Table 2.

**Table 2:** CAZy classification statistics of the genomes of 4 *Akkermansia* isolates.

CAZy-categories	AKK-HX001	AKK-HX002	AKK-HX003	AKK-HX004
Number of AAs	2	2	2	2
Number of CBMs	10	10	9	10
Number of CEs	9	9	9	9
Number of GHs	66	67	65	67
Number of GTs	67	68	69	68
Number of PLs	1	1	1	1

### 3.4 Genomic safety assessment

Safety is a basic, essential characteristic of probiotics. The safety of *A. muciniphila*, a potential next-generation probiotic, is currently controversial. To explore the safety of the 4 *Akkermansia* isolates at the genetic level, they were analyzed for antibiotic resistance and mechanisms, virulence genes, and pathogenicity genes for prediction, respectively.

#### 3.4.1 Antibiotic resistance and mechanism

By comparing the predicted amino acid sequences with the CARD database, the results of AKK-HX001 and AKK-HX002 were consistent, and both of them were annotated with two antibiotic resistance genes (*qacG*, *adeF*), and the antibiotic resistance mechanism was antibiotic efflux pump (resistance-nodulation-cell division, RND; and small multidrug resistance, SMR). On the other hand, AKK-HX003 and AKK-HX004 annotated one more antibiotic resistance gene: *ErmB*, encoding 23S rRNA methyltransferase, whose antibiotic resistance mechanism is related to the change of antibiotic targets (Min et al., 2008) (Table 3). *qacG* is an SMP subclass protein of the SMR family encoded by a plasmid or class I integrator that confers resistance to benzalkonium chloride in bacteria (Heir et al., 1999). *adeF* is involved in the membrane fusion protein encoding the RND efflux pump system, AdeFGH, which equips the bacteria with resistance to fluoroquinolone and tetracycline antibiotics (Coyne et al., 2010). Expression of *ErmB* is inducible by erythromycin. The leader peptide causes attenuation of the mRNA and stabilizes the structure, preventing further translation. When erythromycin is present, it binds the leader peptide causing a change in conformation allowing for the expression of *ErmB* (Min et al., 2008). It renders bacteria resistant to erythromycin, roxithromycin, and lincomycin antibiotics (Hajduk et al., 1999).

**Table 3:** Prediction results of antibiotic resistance genes in 4 *Akkermansia* isolates.

Class definition	Resistance genes	Resistance mechanism	AMR gene family	Antibiotic
AKK-HX001	*qacG*	Antibiotic efflux	SMR	Benzalkonium chloride
	*adeF*	Antibiotic efflux	RND	Fluoroquinolone; tetracycline
AKK-HX002	*qacG*	Antibiotic efflux	SMR	Benzalkonium chloride
	*adeF*	Antibiotic efflux	RND	Fluoroquinolone; tetracycline
AKK-HX003	*ErmB*	Antibiotic target alteration	Erm 23S ribosomal RNA methyltransferase	Streptogramin; streptogramin B; streptogramin A; lincosamide; macrolide
	*qacG*	Antibiotic efflux	SMR	Benzalkonium chloride
	*adeF*	Antibiotic efflux	RND	Fluoroquinolone; tetracycline
AKK-HX004	*ErmB*	Antibiotic target alteration	Erm 23S ribosomal RNA methyltransferase	Streptogramin; streptogramin B; streptogramin A; lincosamide; macrolide
	*qacG*	Antibiotic efflux	SMR	Benzalkonium chloride
	*adeF*	Antibiotic efflux	RND	Fluoroquinolone; tetracycline

#### 3.4.2 Virulent factors

The coding genes of the 4 isolates were annotated in the VFDB database, and the results were consistent, with two virulence factor-coding genes, *katA* and *tufA*, annotated in each strain, accounting for only 0.075–0.080% of the CDS.

#### 3.4.3 Pathogenicity

The PHI annotation results basically corresponded to the virulence gene annotation results, and all 4 isolates were annotated with three pathogenic genes, *katA*, *tufA*, and *fumB*, which accounted for only 0.113–0.121% of the CDS.

### 3.5 Mobile genetic elements analysis

Prokaryotes, such as bacteria, evolve mainly through horizontal gene transfer (HGT) to obtain new genes from other individuals and often produce abundant MGEs in the process (van Dijk, 2020). MGEs can reflect the stability of the strain and its ability to adapt to the environment (Al-Emran et al., 2022), and the 4 isolates were analyzed and found to have MGEs, including genomic islands and the CRISPR-Cas system.

#### 3.5.1 Genomic islands

**Figure 6:**
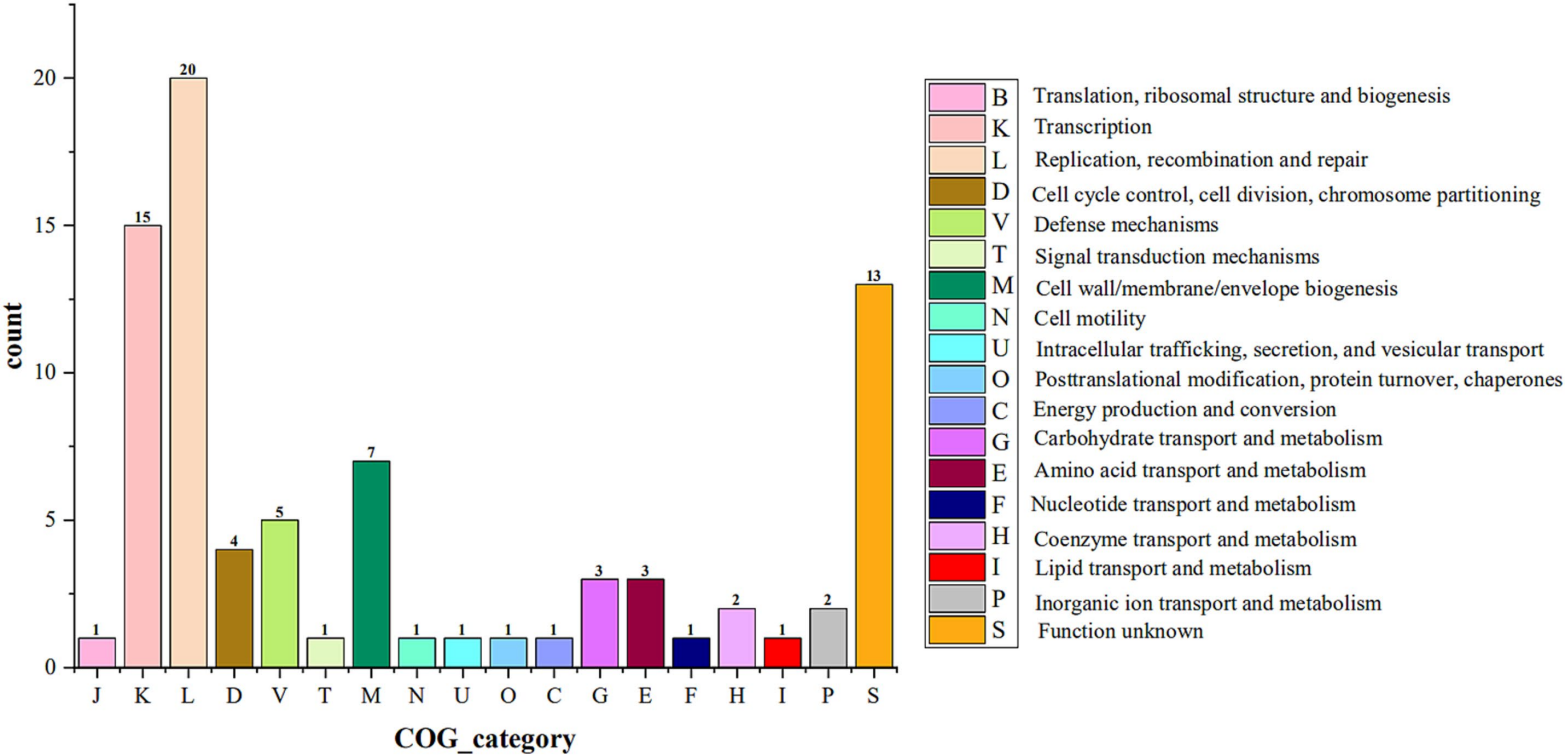
COG categories for the genomes encoding genes on genomic islands of 4 *Akkermansia* isolates.

The 4 isolates had 39, 41, 129, and 91 coding genes involved in constituting 2, 3, 7, and 5 GIs, respectively. COG functional annotation of the above coding genes showed that the coding genes in the GI of the 4 isolates were mainly involved in transcription, replication, recombination and repair processes (Figure 6). To understand the security of the above coding genes at the gene level, they were annotated with CARD, VFDB, and PHI databases, and no other antibiotic resistance genes, virulence factor-coding genes, or pathogenic genes were found, except for one antibiotic resistance gene (*ErmB*) predicted on one GI of AKK-HX004.

#### 3.5.2 CRISPR-Cas system

The results of CRISPR-Cas system prediction showed that AKK-HX001, AKK-HX002, and AKK-HX004 each had a complete IC-based CRISPR system, including cas2, cas1, cas4, cas3, cas5c, cas8c, and cas7c proteins. AKK-HX003 was predicted to have only 4 CRISPRs; no cas proteins were found, and no complete CRISPR-Cas system (Table 4).

**Table 4:** Prediction results of the CRISPR-Cas system of 4 *Akkermansia* isolates.

Class definition	AKK-HX001	AKK-HX002	AKK-HX003	AKK-HX004
Number of CRISPR	4	5	4	4
Number of Cas cluster	1	1	–	1
Cas type	CAS-TypeIC	CAS-TypeIC	–	CAS-TypeIC
Number of cas gene	7	7	–	7
Cas gene	cas2_TypeI-II-III cas1_TypeIC cas4_TypeI-II cas3_TypeI cas5c_TypeIC cas8c_TypeIC cas7c_TypeIC	cas2_TypeI-II-III cas1_TypeIC cas4_TypeI-II cas3_Type cas5c_TypeIC cas8c_TypeIC cas7c_TypeIC	–	cas2_TypeI-II-III cas1_TypeIC cas4_TypeI-II cas3_Type cas5c_TypeIC cas8c_TypeIC cas7c_TypeIC

### 3.6 Secondary metabolite gene cluster prediction

Probiotic strains have to be competitively colonized and survive in the gut, and bacteria with the ability to produce bacteriocins are more advantageous (Heo et al., 2021).

By comparison with the antiSMASH database, two clusters of secondary metabolite genes were predicted for all 4 isolates, both representing terpenoids (Table 5).

**Table 5:** Characteristics of gene clusters in 4 *Akkermansia* isolates.

Class definition	AKK-HX001	AKK-HX002	AKK-HX003	AKK-HX004
Region	Region 1.1	Region 3.1	Region 7.1	Region 4.1
	Region 7.1	Region 6.1	Region 13.1	Region 6.1
Region position	569,022–589,942	309,741–330,634	229,105–250,025	401,871–422,764
	1–20,664	9,058–29,978	90,731–111,624	31,644–52,564
Type	Terpene	Terpene	Terpene	Terpene

## 4 Discussion

NGPs are suitable for conventional food or dietary supplements and pharmaceutical applications. It offers a variety of advantages over traditional probiotics, including opportunities for personalized probiotic therapies, involvement in synthetic biology and gene editing, participation in combination therapies, targeted delivery methods, and application in therapeutic settings (Abouelela and Helmy, 2024; Lalowski and Zielińska, 2024). *A. muciniphila*, a member of the next generation of probiotics, has received much attention *in vivo.* However, due to its harsh requirements for the growth environment, the acquisition of its isolates has become a formidable challenge, which has hindered the study of the genomes of *Akkermansia* isolates. Currently, studies on the genome of *Akkermansia* have mainly come from the analysis of gastrointestinal macro-genome assembly data. Therefore, in order to fill part of the gap of the genomic data of *Akkermansia* isolates as much as possible, the present study was carried out to analyze the genomes of 4 *Akkermansia* isolates obtained from the preliminary isolation in our laboratory, and revealed their genomic features, potential genomic functions, and also evaluated their safety and security at the genetic level.

Whole genome sequencing results showed that the genome size and CDS counts of the 4 *Akkermansia* isolates were much higher than the type strain DSM22959, suggesting that there might be a substantial difference between the genomes of our 4 isolates and the type strain. The GC content usually reflects the stability of the genome, and a high GC content may lead to the organisms consuming more energy during the replication process (Kandasamy et al., 2022). Whereas the results indicated that the GC content of these 4 isolates was higher than that of the model strain, suggesting that there may be some differences between them and the model strain in terms of metabolism. Homology analysis of the 4 isolates displayed that each possessed a different number of specific genes, given that most of these specific genes were hypothetical proteins that were not annotated to their corresponding functional classifications, we could not analyze them further. However, the presence of specific genes corroborates the diversity of the *Akkermansia* genome to a certain extent.

Phylogenetic analysis based on 16S rRNA and phylogenomic analysis based on single-copy core genes disclosed that these 4 isolates did not belong to the same phylogroup as the type strain. Phylogenetic analysis based on 16S rRNA revealed that AKK-HX001 belonged to a different branch from the other three strains. In contrast, the phylogenomic analysis based on single-copy core genes more finely classified AKK-HX002 and AKK-HX004 into the same branch, while AKK-HX003 was classified in the same branch as AKK-HX001. As for the reason why AKK-HX001 and AKK-HX003 were in a different evolutionary branch from the rest of the strains, we hypothesized that it is host-associated. The 4 isolates came from different human hosts, whose habits, such as living and eating, may have influenced their genotypes and phenotypes, which in turn induced their evolution. Functional annotation of the genomes of the four isolates also revealed that AKK-HX001 and AKK-HX003 were different from the other two strains to a different extent, and the presence of a higher number of unique genes in AKK-HX001 and AKK-HX003 may also contribute to their evolutionary differences. This further confirmed that phylogenomic analysis may be more rigorous than based on 16S rRNA phylogenetic analysis when full genomes are available. This classification result was also confirmed in the genome average nucleotide concordance. The ANI values between the 4 isolates and DSM22959 were 87.49, 87.48, 87.32, and 87.41%, respectively. Based on the threshold ANI > 96% between prokaryotic species (Richter and Rossello-Mora, 2009), it can be assumed that the 4 isolates are not of the same species of *Akkermansia* as DSM22959. Rather, the ANI values between the 4 isolates were as high as 98.53–99.93%, and it can be assumed that they belong to the same species within the genus *Akkermansia*. This further confirmed that differences exist with respect to the genomes of the 4 isolates and the model strain.

COG functional annotation is a relatively simple and reliable way to identify potential orthologs and paralogs based on complete genomic protein sequences (Galperin et al., 2019). The KEGG database is a large-scale integrated repository of systematic, genomic, chemical, and health information (Kanehisa et al., 2023). KEGG is based on the annotation of the functional ortholog list, which connects genomic information with higher-order functional information, stored in the GENES and KEGG pathway databases, providing rich pathway information and helping us understand the biological functions of genes systematically (Kanehisa and Goto, 2000). COG analysis of the predicted coding genes of the 4 isolates indicated that most of the genes were assigned to the functional categories of information storage and processing, cellular signaling, and metabolism, which are frequently associated with bacterial life activities and energy metabolism, suggesting that they possessed a strong ability to metabolize amino acids and carbohydrates. Immediately following the KEGG database annotation of the coding genes, similar results were obtained, with the vast majority of genes assigned to metabolism (especially carbohydrate metabolism) and genetic information processing pathways, further corroborating the importance of carbohydrates as an energy source in the biological functions of *Akkermansia* strains. The carbohydrate-active enzyme studies of the 4 isolates will hopefully deepen our insights into their growth and metabolism mechanisms. CAZy is a database resource on enzymes capable of synthesizing or breaking down complex carbohydrates and sugar complexes, categorizing carbohydrate-active enzymes into different protein families based on amino acid sequence similarities in the structural domains of the proteins (Cantarel et al., 2009). The database supplies classification and related information on enzymes for synthesizing, metabolizing, and transporting carbon compounds (Lombard et al., 2014). Annotation of the CAZy database identified the 4 isolates as being rich in genes coding for carbohydrate-active enzymes (CAZymes), mainly GHs and GTs. GHs hydrolyze glycosidic bonds between two or more carbohydrates, or between carbohydrates and non-carbohydrate fractions, and the genes of the GHs annotated this time belonged to the GH2, GH20, and GH29 families, which are the enzymes with *β*-galactosidase activity, β-N-acetylamino glucosidase and β-N-acetyl galactosidase activity, and *Α*-L-fucosidase activity, respectively. These enzymes are sufficiently required for *A. muciniphila* to fully utilize 2′-fucosyl lactose (Lawson et al., 2020). GTs are enzymes that catalyze the formation of glycosides from glycosidic bonds, and the 4 isolates annotated with GTs were mainly derived from the GT2 and GT4 families, all of which possessed multiple glycosyltransferase activities (Cantarel et al., 2009). Carbohydrate-active enzymes digest most of our complex pool of dietary polysaccharides, ultimately producing short-chain fatty acids, which are involved in numerous physiological and biochemical processes in our body (Wong et al., 2006). Several clinical studies have suggested that short-chain fatty acids may bridge the involvement of *A. muciniphila* in glucose metabolism (Palmnäs-Bédard et al., 2022), a role that our CAZy annotation results seem to echo, providing a gene-level explanation for.

A review described the role of *A. muciniphila* in reducing intestinal inflammation, modulating immune response, and enhancing intestinal barrier function, supporting its potential as a probiotic (Ioannou et al., 2024). A recent study also validated the safety of *A. muciniphila* for use as a probiotic (Lv et al., 2024). Since *Akkermansia* is a little-studied genus and its mechanism of action is not well understood, it is difficult to analyze it directly at the species level, which should be studied at the strain level (Hill et al., 2014). A significant need exists to explore the antibiotic resistance genes, virulence genes, and pathogenicity genes of these 4 isolates of *Akkermansia* at the genetic level to assess their safety. Antibiotic resistance mechanisms include intrinsic resistance, acquired resistance, and adaptive resistance, among which intrinsic and acquired resistance are closely related to the strain’s antibiotic resistance genes. Therefore, the comparison between intrinsic antibiotic resistance genes and adaptive resistance genes should be emphasized (Ogawara, 2019). Intrinsic antibiotic resistance genes may mediate nonspecific resistance mechanisms, including nonspecific efflux pumps and inactivating enzymes, whereas acquired antibiotic resistance genes can arise from mutations and/or horizontal transfer of genes that mediate specific resistance mechanisms, including enzymes modifying the antibiotic or the antibiotic target (Peterson and Kaur, 2018). In the present study, a total of three antibiotic resistance genes were annotated in the 4 isolates, among which the *adeF* and *ermB* genes conferred multiple antibiotic resistances to the bacteria, and were hypothesized to mediate nonspecific resistance mechanisms, potentially belonging to intrinsic resistance genes. This is consistent with the report of Filardi et al. that the *adeF* gene is widely present in the genome of *Akkermansia* strains (Filardi et al., 2022). The *qacG* gene confers bacterial benzalkonium chloride (quaternary ammonium disinfectant) resistance and may mediate specific resistance mechanisms; however, available reports on *Akkermansia* have not identified such a gene for this resistance. It is worth mentioning that in our unpublished study on antibiotic susceptibility, the 4 isolates showed resistance to glycopeptides, aminoglycosides, and other antibiotics, but the corresponding resistance genes were not found in this genomic analysis. The reasons for this may involve several factors: firstly, *A. muciniphila* is a Gram-negative bacterium, which makes it intrinsically resistant to glycopeptides due to its outer membrane (Gauba and Rahman, 2023). In addition, aminoglycosides are known to be less active against Gram-negative anaerobes because energy is required for this process, which is mostly unavailable due to their metabolism (Sood et al., 2023). Therefore, it is not surprising that there is genotypic and phenotypic inconsistency in antibiotic resistance in *Akkermansia* strains. The predictions of virulence and pathogenicity genes were more aligned, with all 4 isolates predicting the *katA* gene, which is associated with stress survival, and the *tufA* gene, which is associated with adhesion ability. Among these genes, the *katA* gene encodes catalase, which helps bacteria resist reactive oxygen species (Weber et al., 2004), and belongs to the category of unaffected pathogenicity, which is associated with host opportunistic pathogenic infections (Xia et al., 2020). Su et al. showed that *Pseudomonas aeruginosa* (PA) exhibited higher *katA* expression under anaerobic conditions compared to aerobic conditions and acted as a protector during anaerobic respiration in PA (Su et al., 2014), suggesting that the ability of *A. muciniphila* to tolerate trace amounts of oxygen may be attributed to *katA*. *tufA* encodes the elongation factor Tu (EF-Tu), which is involved in the binding of aminoacyl-tRNA to the ribosomal A site (Rodnina et al., 2017). Annotation results suggest that its mutant phenotype belongs to unaffected pathogenicity and/or increased virulence, associated with skin infection, food poisoning, and respiratory disease (McLean et al., 2019). A *fumB* pathogenic gene, encoding fumarate hydratase class I, the absence of which causes *Salmonella typhimurium* to exhibit lower virulence, was also annotated (Noster et al., 2019). The presence of several of these virulence factors may result in a pathogenic risk for the strain, but several previous animal experiments (Depommier et al., 2020; Wang et al., 2020) and clinical trials (Depommier et al., 2019; McMurdie et al., 2022) have demonstrated that *A. muciniphila* is safe. Geerlings et al. (2018) showed that it is already present within the first year of life, accounting for 3–5% of the gut microbiota, and gradually increasing with age until adulthood (Collado et al., 2007). Since its safety has been controversial, we favor the presence of these virulence factors to aid in its adaptation to the environment and facilitate colonization.

In addition, to assess the intergenomic transfer of antibiotic resistance genes, virulence genes, and pathogenic genes, as well as resistance to invasion by exogenous genetic material, 4 isolates were predicted to have MGEs (GIs, CRISPR-Cas system). GIs are the most significant class of HGT, carrying genes affecting the host pathobiology and providing a selective advantage for host adaptation (Novick and Ram, 2016). Four isolates were each predicted to have variable numbers of GIs, and no virulence factors were identified in GI-encoding genes except for one *EmrB* resistance gene. They were mostly associated with genetic information storage and processing. All isolates except AKK-HX003 were matched with the detection of an IC-type CRISPR-Cas system, which is most typical of *Akkermansia* strains (Karcher et al., 2021). The absence of CRISPR sites may enhance the stability of the strain’s genome and improve its adaptability to the environment (Al-Emran et al., 2022). Therefore, it can be assumed that the 4 isolates have a low risk of potential transfer and some resistance to invasion by exogenous genetic material.

Terpenes, a general term for a range of terpenoids, many of which have important biological activities, were identified in a previous study as terpene gene clusters in the genus *Pseudovibrio* with the potential to produce novel active compounds (Naughton et al., 2017). Two terpene gene clusters were identified in each of the 4 isolates in this study, but were not compared to a particular class of terpene compounds known in databases, probably because little research has been done on *Akkermansia* secondary metabolite gene clusters and due to the high percentage of genes encoding hypothetical proteins in the genome of this genus, suggesting a high potential for mining the functions of its genome.

## 5 Conclusion

This study, involving whole genome sequencing and genome analysis of 4 isolates of *Akkermansia*, resulted in a striking similarity between their genomes, with only about 87% ANI similarity to the model strain DSM22959. Additionally, the 4 isolates did not belong to the same phylogroup as DSM22959. The 4 isolates also showed homogeneity in terms of genome function, being mainly involved in the processing of genetic information, metabolic processes, and carrying abundant carbohydrate-active enzymes. Although a small number of antibiotic resistance genes, virulence genes, and pathogenicity genes were annotated, it was almost proven that these genes were not associated with pathogenicity. No significant mobile genetic elements were detected, indicating a low potential risk of gene transfer and a certain level of safety at the genetic level. In contrast to the relatively well-studied *Lactobacillus* and *Bifidobacterium* species, *Akkermansia* exhibited a certain number of annotated metabolic genes, albeit slightly fewer than those of the former two, suggesting that its probiotic effects may not be entirely metabolism-dependent. Additionally, a significant proportion of the genes in the four *Akkermansia* isolates were annotated as “Function Unknown,” highlighting substantial gaps in our understanding of the *Akkermansia* genome. This observation indirectly suggests that *Akkermansia* possesses considerable potential for further exploration, with these uncharacterized genes potentially holding the key to elucidating its probiotic role. Therefore, it is imperative to isolate and analyze additional strains to enhance genomic data and facilitate the functional characterization of these “Function Unknown” genes.

## Data Availability

The whole genome sequence datasets analyzed for this study can be found in the NCBI GenBank with the accession numbers JBGKAZ000000000, JBGKBA000000000, JBGKBB000000000, and JBGKBC000000000.
