# Supplementary material for: Whole-genome sequencing and genomic analysis of four Akkermansia strains newly isolated from human feces
Source: Front Microbiol. 2024 Dec 16;15:1500886. doi: 10.3389/fmicb.2024.1500886 (PMC11683593; doi:10.3389/fmicb.2024.1500886)
Supplement: Supplementary file 1 [file Supplementary_file_1.docx]

Supplementary Material

## Supplementary Figures


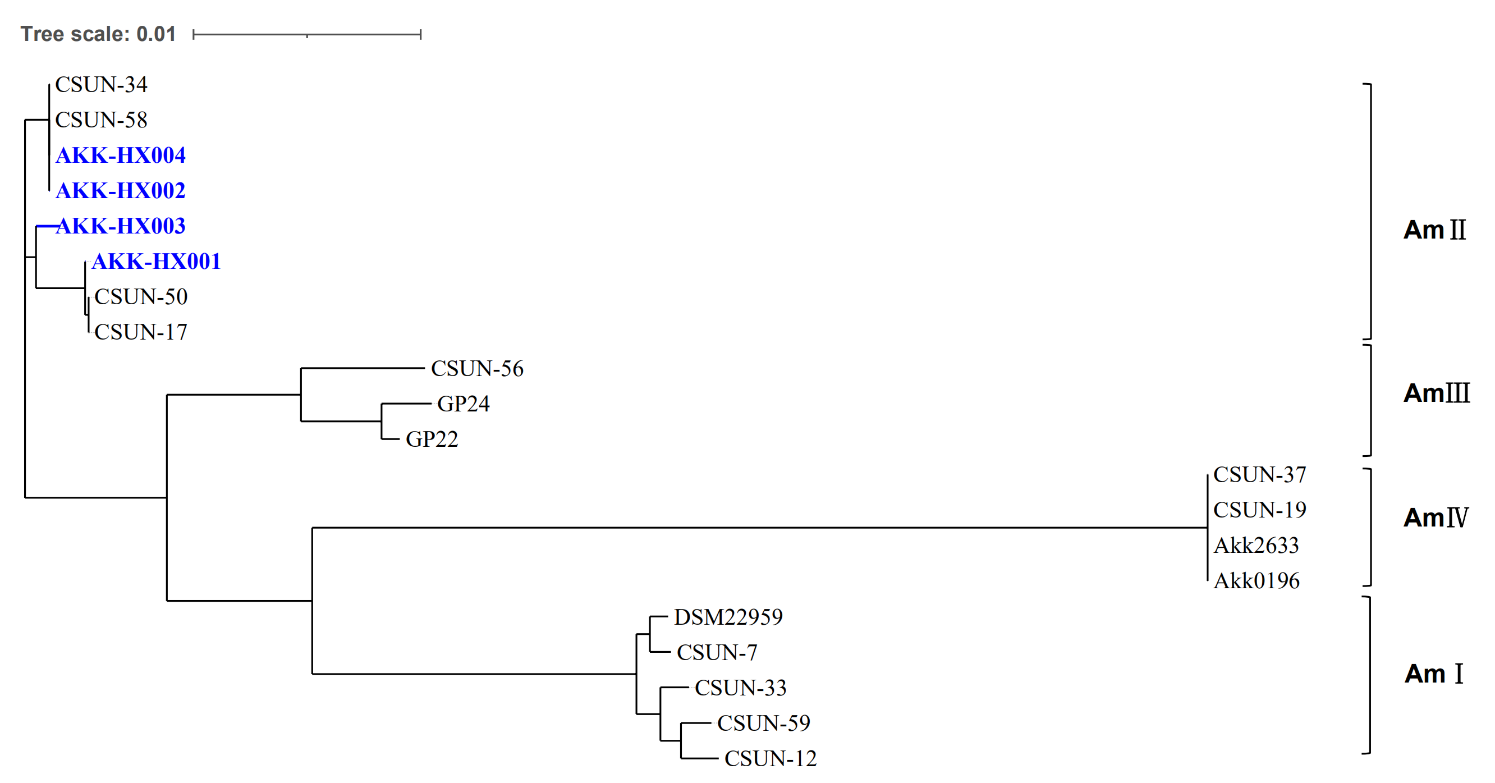


**Supplementary Figure 1.** The phylogenetic tree based on single-copy core genes.
